# Integrated Transcriptomic, Enzymatic, and Immunolocalization Analysis Reveals Pectin Remodeling-Mediated Defense Against *Fusarium oxysporum* f. sp. *cubense* Race 4 in Banana

**DOI:** 10.3390/cimb48080847

**Published:** 2026-08-20

**Authors:** Rahat Sharif, Yanqing Xing, Huimin Song, Hangbo Cao, Yu Li, Wenzheng Liu, Huiling Zhan, Chunxiang Xu

**Affiliations:** Department of Pomology, College of Horticulture, South China Agricultural University, Guangzhou 510642, China

**Keywords:** *Musa acuminata*, *Fusarium oxysporum* f. sp. *cubense*, pectin degradation, damage-associated molecular patterns, disease resistance

## Abstract

*Fusariumoxysporum* f. sp. *cubense* race 4 (*Foc* 4) causes Fusarium wilt by penetrating root cell walls, yet the molecular basis of cell wall-mediated resistance remains poorly understood. Here, we investigated the transcriptional, enzymatic, and cellular responses of the resistant banana cultivar Dongjiao No. 1 (DJ) and its susceptible mutant *ke2* following *Foc* 4 infection. RNA sequencing revealed that DJ specifically upregulated a pectin degradation cassette comprising pectin methylesterase (*PME3-Like*), pectin acetylesterase (*PAE1*), and polygalacturonases (*PG1*, *PG3*, *PG5*, *PG-Like-4*) at the bud seedling stage. Immunolocalization further revealed robust, tissue-specific PME deployment, with stable abundance at the primary infection site and differential redistribution in aerial tissues. This enzymatic cascade degraded homogalacturonan, confirmed by the simultaneous loss of pectin epitopes recognized by JIM5 and JIM7 antibodies. Additionally, the upregulation of *PTI1*, MAPK cascades, calcium-dependent protein kinases (*CDPK3*, *CML31*), and respiratory burst oxidase homologs (RBOHs) was also observed in DJ. The differential transcription of salicylic acid signaling (TGA1–PR1), jasmonic acid derepression (TIFY/JAZ), and flavonoid phytoalexin biosynthesis in DJ further reinforced the defense response. In contrast, *ke2* failed to activate the pectin degradation machinery, exhibited attenuated immune signaling, and retained intact pectin vulnerable to pathogen exploitation. These findings establish pectin degradation-mediated immunity as a resistance mechanism in banana and provide potential targets for Fusarium wilt resistance breeding.

## 1. Introduction

Bananas (*Musa* spp.), perennial herbaceous plants belonging to the family Musaceae, rank among the most important fruit crops cultivated throughout tropical and subtropical regions worldwide [1,2]. As the fourth most important global staple food after rice, wheat, and maize, they constitute a cornerstone of food security and household income in many low-income, food-deficient nations [3]. Regions with expansive tropical climates, such as South China, are particularly suitable for large-scale cultivation and consumption. However, global banana production faces a severe and escalating threat from Fusarium wilt, a devastating disease caused by the soil-borne fungus *Fusarium oxysporum* f. sp. *cubense* (*Foc*) [3]. The emergence of the highly virulent Tropical Race 4 (*Foc* 4) is especially alarming, as it can infect nearly all commercial cultivars [4]. Following entry through root wounds or natural openings, the pathogen colonizes the vascular system [5,6]. It then proliferates within the xylem vessels, obstructing the transport of water and nutrients, a process that ultimately leads to wilting and plant death [4].

Banana roots exhibit a complex anatomical architecture comprising the rhizodermis, exodermis, cortical parenchyma, endodermis, pericycle, and central vascular cylinder (xylem and phloem) [7,8], which collectively function as sequential physical barriers against soil-borne vascular pathogens. Following entry through root wounds or natural openings, *Foc* 4 progresses intercellularly through the cortex, breaches the endodermis and pericycle, and systemically colonizes the xylem vessels, where its proliferation induces vascular occlusion, disrupts water and nutrient transport, and ultimately leads to wilting and plant death [8]. Pectin, a major matrix polysaccharide in the middle lamella and primary cell walls of cortical and xylem parenchyma cells, is essential for maintaining cell adhesion, wall integrity, and tissue impermeability [9]. Given that *Foc* 4 relies on cell wall-degrading enzymes to penetrate these barriers and establish systemic infection [4,10], the structural status and dynamic remodeling of pectin within xylem-associated tissues represent critical determinants that govern whether the pathogen is restricted at the root cortex or achieves successful vascular colonization.

The plant cell wall (PCW) represents the primary physical barrier against pathogen invasion, a function critically dependent on pectin as a fundamental structural component [11]. As a complex matrix polysaccharide abundant in the primary cell wall and middle lamella, pectin comprises galacturonic acid-rich domains, including homogalacturonan (HG) and rhamnogalacturonans I and II (RG-I and RG-II), which collectively maintain cell wall integrity and intercellular adhesion [12]. This structural organization further enables pectin to act as a ligand for wall integrity sensors, allowing plants to monitor mechanical status and mount defense responses upon pathogen-mediated degradation [12]. Phytopathogens circumvent this barrier by secreting cell wall-degrading enzymes (CWDEs), with pectin methylesterases (PMEs) launching a critical early attack through the demethylation of HG chains. This modification enhances wall porosity and facilitates the subsequent action of other pectin-degrading enzymes [13,14]. In response, plants counteract through cell wall reinforcement and the production of specific PME inhibitors (PMEIs). Disease resistance is tightly associated with the dynamic regulation of pectin methylesterification during infection, governed by the interplay among PME activity, PMEI levels, and the degree of methylesterification (DM) and structural patterning of HGs [9,15,16]. Extensive wall degradation liberates oligogalacturonides (OGs), which function as damage-associated molecular patterns (DAMPs) to activate immune responses, including the transcriptional reprogramming of defense-related genes [17]. PME genes occupy a central position in this regulatory framework, modulating enzymatic activity upon perception of environmental stimuli, notably pathogen infection [18].

Pectin remodeling as a defense strategy is evolutionary conserved across diverse crop species confronting vascular wilt pathogens [19,20,21]. In banana, the interaction with *Foc* triggers substantial remodeling of the cell wall architecture [4,22], yet the precise regulatory network governing pectin methylesterification and its contribution to resistance or susceptibility remains incompletely understood. Therefore, investigating the spatiotemporal expression patterns and functional roles of PME-related genes during pathogen colonization is essential for deciphering the molecular basis of banana–*Foc* interactions and for developing durable strategies against Fusarium wilt.

We hypothesized that resistance to *Foc* 4 in banana is mediated by an infection-induced, coordinated pectin degradation cascade in root cortical and xylem parenchyma cells. Herein, we employed the resistant banana cultivar Dongjiao No. 1 (DJ) and its susceptible mutant *ke2* to investigate the transcriptional reprogramming elicited by *Foc* 4 infection. As *Foc* 4 is a vascular pathogen that systemically colonizes the plant, leaf transcriptomics were employed to capture systemic defense responses, while root tissues were used for enzymatic and cellular validation of pectin remodeling at the primary infection site. RNA sequencing revealed substantial differential expression of PME and PG genes between DJ and *ke2*, alongside significant enrichment of plant hormone signal transduction, plant–pathogen interaction, flavonoid biosynthesis, and pectin metabolism pathways. Subsequent analysis identified numerous differentially expressed genes (DEGs) within these pathways, a considerable proportion of which were functionally linked to pectin dynamics. To complement these transcriptomic findings, we examined the PME and PG activity and epitope recognition patterns of pectin-specific antibodies in banana roots and leaves. Collectively, our study provides novel mechanistic insights into how pectin remodeling regulates banana defense responses against *Foc* 4.

##  2. Materials and Methods 

### 2.1. Plant Materials

Experiments were conducted using the disease-resistant banana cultivar ‘Dongjiao No. 1’ (DJ; *Musa* spp., AAA group) and its susceptible mutant *ke2* [23] at three developmental stages: tissue culture (TC), bag seedling stage (BSS), and shooting stage (SS, corresponding to the inflorescence-emerging stage). For the TC stage, sucker shoot tips were surface-sterilized and proliferated on Murashige and Skoog (MS) [24] medium supplemented with 6-benzylaminopurine (BA; 4 mg/L) and α-naphthaleneacetic acid (NAA; 0.2 mg/L). Shoots were subsequently rooted on solid MS medium containing indole-3-butyric acid (IBA; 0.5 mg/L) and NAA (0.2 mg/L). Uniform plantlets bearing 3–4 leaves were selected and pre-cultured in liquid rooting medium on a rotary shaker (84 rpm) for two weeks prior to inoculation.

For the BSS stage, rooted plantlets from solid medium were acclimatized at room temperature for one week and then transplanted into a sterilized potting mix in 30-cell trays. After 4–6 weeks under a 12 h light/dark photoperiod at controlled temperatures, seedlings were transferred to 200 mm pots and maintained in a physical containment glasshouse at approximately 26 °C under natural daylight. Plants were inoculated when they reached approximately 20 cm in height.

For the SS stage, plants were grown in either a healthy field or a banana Fusarium wilt germplasm identification plot that had been artificially inoculated with *Foc* 4. Roots were sampled at the inflorescence-emerging stage (the peak period for Fusarium wilt field symptoms). Nylon mesh was used to guide root growth to facilitate collection of clean root tissues.

### 2.2. Inoculation of a Pathogen

The *Foc* 4 isolate was cultured on solid potato dextrose agar (PDA; 200 g/L potato, 16 g/L sucrose, 13 g/L agar) in darkness for seven days, after which it was transferred to liquid yeast peptone dextrose (YPD; 10 g/L yeast extract, 20 g/L peptone, 20 g/L dextrose) medium to induce spore production. Conidia were harvested after five days of incubation on a rotary shaker at 100 rpm. At the TC stage, a modified wounded-root immersion method was adopted. After two weeks in liquid rooting medium, one root per plantlet was wounded (cut approximately 1 cm from the root base), and the plantlet was transferred to liquid rooting medium containing *Foc* 4 at a final concentration of 5 × 10^2^ conidia/mL. Control plantlets were treated identically but with sterile distilled water. Samples were collected at 24 h post-inoculation. At the BSS stage, banana seedlings at approximately 20 cm height were inoculated using a modified wounded root irrigation method. In brief, four shallow vertical cuts (approximately 2 cm deep) were made around the root system of each seedling using a sterile scalpel, followed by the application of 30 mL of a *Foc* 4 spore suspension (1 × 10^6^ conidia/mL). Control plants received the same volume of sterile distilled water applied to similarly wounded roots. At the SS stage, plants grown in the artificially inoculated identification plot served as the infected treatment, while plants from the healthy field served as controls. The inoculation and sampling workflow is outlined schematically in Figure S1. Each treatment consisted of three biological replicates, with ten seedlings per replicate for the TC and BSS stages [25].

### 2.3. Total RNA Extraction and RNA-Sequencing Analysis

Total RNA was extracted from banana leaf tissues using the RNAprep Pure Plant Kit (Tiangen, Beijing, China) and reverse-transcribed with HiScript II Q RT SuperMix (Vazyme, Nanjing, China) [26]. Leaf tissues were selected for transcriptomic analysis to capture systemic defense responses to the vascular pathogen *Foc* 4. Root tissues were used for subsequent enzymatic and immunolocalization assays to validate cell wall remodeling at the primary infection site. A 1 μg aliquot of total RNA was used for mRNA enrichment with poly-T magnetic beads. First-strand and second-strand cDNA were then synthesized, and the resulting overhangs were converted to blunt ends through exonuclease/polymerase activity. Following adenylation of the 3′ ends, NEBNext adapters (Ipswich, MA, USA) featuring hairpin loop structures were ligated. The sequencing library was purified using AMPure XP beads (Brea, CA, USA) and its quality assessed with an Agilent Bioanalyzer 2100. Sequencing was carried out on an Illumina Novaseq 6000 (San Diego, CA, USA) platform to generate raw reads. These raw reads were processed using the BMK Cloud bioinformatics pipeline, Beijing, China (https://www.biocloud.net).

RNA-seq data quality was assessed using FastQC Cambridge, UK (v0.11.9). As summarized in Table S1, the 12 libraries generated between 45,094,750 and 56,880,096 raw reads per sample. After adapter trimming and quality filtering, clean reads ranged from 44,168,906 to 55,007,426, corresponding to 6.63–8.25 Gb of clean bases per library and an average raw-to-clean retention rate of approximately 97%. The per-base error rate was 0.02% across all samples. Quality scores were high, with Q20 values exceeding 96.36% and Q30 values exceeding 90.97% in every library. GC content ranged from 50.31% to 51.13%, consistent with the expected nucleotide composition of Musa acuminata transcriptomes. These metrics collectively confirmed the high fidelity and depth of the sequencing data, supporting their suitability for alignment, quantification, and differential expression analysis.

### 2.4. DEGs and Gene Ontology (GO)/KEGG Enrichment Analysis

We characterized the assembled genes by conducting BLAST homology searches against the NCBI, Swiss-Prot, GO, Pfam, and KEGG databases. Gene expression levels were quantified as fragments per kilobase of transcript per million mapped reads (FPKM), and differentially expressed genes (DEGs) were identified using thresholds of |log_2_FC| ≥ 2 and FDR < 0.05. For integrated visualization of defense-related transcriptional reprogramming across all genotype–treatment combinations, we constructed a curated gene panel from the union set of DEGs that satisfied the significance threshold (|log_2_FC| ≥ 2, FDR < 0.05) in at least one of the four pairwise comparisons (DJCK vs. *ke2*CK, *ke2*I vs. *ke2*CK, DJI vs. DJCK, and DJI vs. *ke2*I). For data visualization, we created heatmaps with TBtools-II [27] and plotted GO enrichment results using the web-based platform provided by NEWCORE biotech (Shanghai, China; https://www.bioinformatics.com.cn/ (accessed on 15 March 2026).

### 2.5. PME and PG Activity Assay

PME activity was determined according to the method described by [22] with slight modifications. Root and leaf tissues were analyzed separately. The assay mixture contained citrus pectin as the substrate in an appropriate reaction buffer. The reaction was initiated by the addition of crude enzyme extract, and PME activity was quantified based on the rate of demethylation, as determined by the release of free carboxyl groups. One unit (U) of PME activity was defined as the amount of enzyme required to release 1 μmol of carboxyl groups per minute under the assay conditions. Polygalacturonase (PG) activity was measured according to the methods described by [28]. The reaction was carried out in a buffer system containing polygalacturonic acid as substrate. Following incubation, the liberated reducing sugars were quantified spectrophotometrically. One unit (U) of PG activity was defined as the amount of enzyme releasing 1 μmol of reducing sugar per minute under the assay conditions.

### 2.6. Immunofluorescence Labeling and Quantification of the Signal Intensity

Fluorescence fixation and immunolabeling were performed as described by [29]. Primary antibodies, JIM5 and JIM7, were from Complex Carbohydrate Research Center (Athens, GA, USA). Quantitative and fluorescence analyses were done with ZEN 2.3 (Oberkochen, Germany) software and an Axio Imager D2 microscope (Carl-Zeiss) Oberkochen, Germany, respectively. Three biological replicates per treatment were assessed. Fluorescence intensity was measured as integrated density using uniform signal thresholds per region of interest, with consistent epitope-specific thresholds maintained across treatments and replicates. Fresh samples were used for immunofluorescence to evaluate signals and epitope levels.

### 2.7. Statistical Analysis

All statistical evaluations were carried out utilizing IBM SPSS Statistics version 26.0 (IBM Corporation, Armonk, NY, USA). Experiments were conducted in at least three replicates, with results expressed as mean ± standard error (SE). Student’s *t*-test was applied to compare the two cultivars, and ANOVA was utilized for broader statistical analysis.

## 3. Results

### 3.1. Overview of DEGs

**Figure 1:**
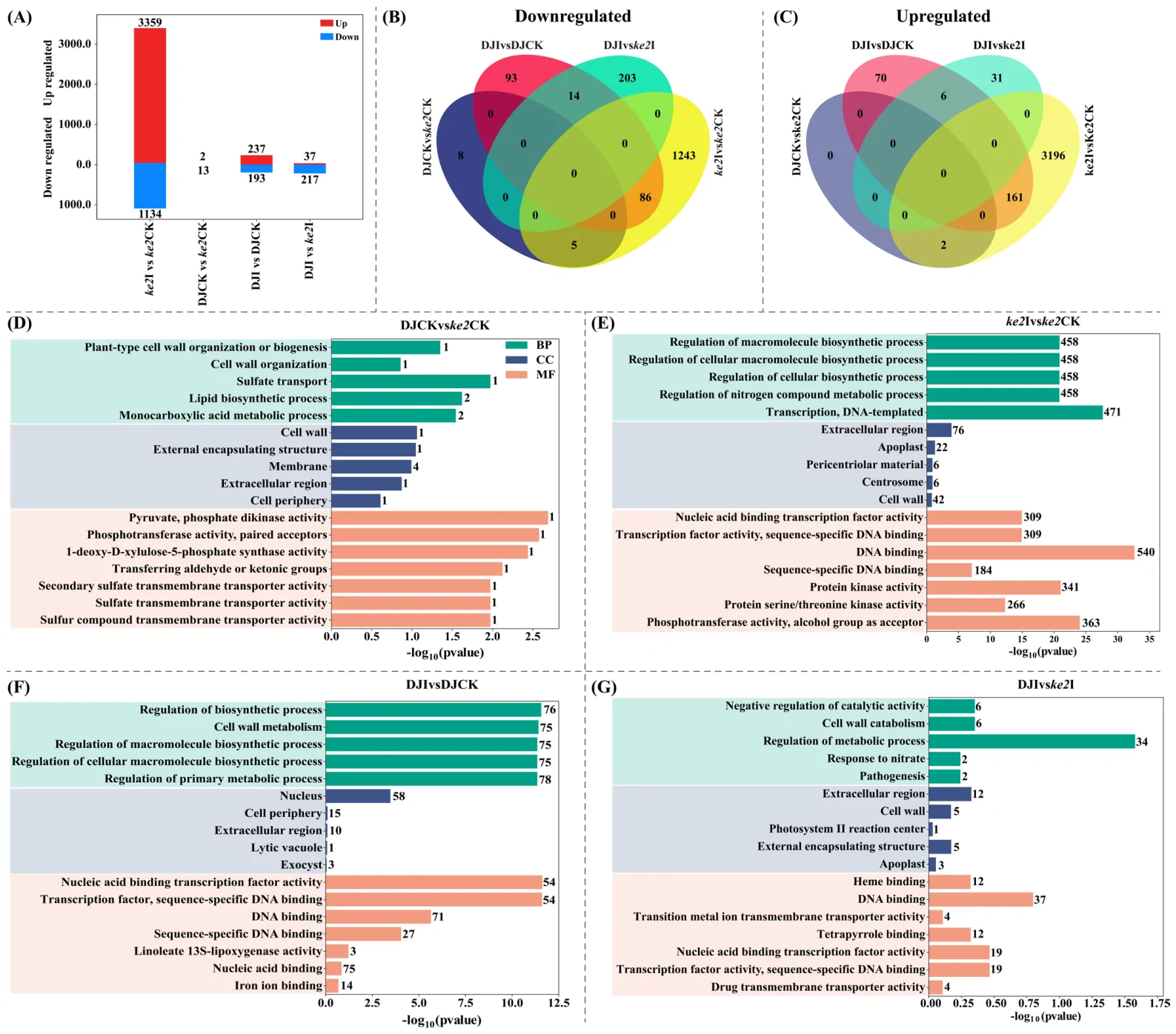
Analysis of differentially expressed genes (DEGs) and Gene Ontology (GO) enrichment. (**A**) Bar chart showing the number of upregulated (red) and downregulated (blue) genes across four comparisons. The y-axis is presented on a logarithmic scale. (**B**,**C**) Venn diagrams illustrating the overlap of downregulated (**B**) and upregulated (**C**) genes. (**D**–**G**) GO enrichment analysis of DEGs for (**D**) DJCK vs. *ke2*CK, (**E**) *ke2*I vs. *ke2*CK, (**F**) DJI vs. DJCK, and (**G**) DJI vs. *ke2*I. Enriched terms are categorized into Biological Process (BP, green), Cellular Component (CC, blue), and Molecular Function (MF, salmon). The x-axis represents the significance of enrichment as −log_10_ (*p*-value). DJ, Dongjiao No. 1 (resistant); *ke2*, susceptible mutant; CK, control (uninoculated); I, infected with *Foc* 4.

Prior to differential expression analysis, quality control of the 12 RNA-seq libraries confirmed robust sequencing metrics, including clean read counts of 44.17–55.01 million per sample, error rates of 0.02%, Q20 > 96.36%, Q30 > 90.97%, and GC contents of 50.31–51.13% (Table S1), indicating that the dataset was of high quality and adequate for downstream transcriptomic comparisons. To elucidate the differential transcriptional responses between DJ and *ke2* to *Foc* 4 infection, RNA-seq analysis was performed. At the basal level, the two cultivars exhibited minimal transcriptional divergence, with only 15 DEGs identified between the uninfected controls (DJCK vs. *ke2*CK), comprising merely 2 upregulated and 13 downregulated genes (Figure 1A). This extremely small pool indicates that resistance in DJ is not established through constitutive transcriptional activation before infection. Upon pathogen challenge, the susceptible mutant *ke2* mounted the most extensive transcriptional reprogramming (*ke2*I vs. *ke2*CK), with 3359 upregulated and 1134 downregulated genes. The resistant cultivar DJ showed a comparatively moderate response (DJI vs. DJCK), with 237 upregulated and 193 downregulated genes. In the direct comparison between infected genotypes (DJI vs. *ke2*I), 37 genes were upregulated, and 217 were downregulated in DJ relative to *ke2*I, reflecting the divergent infection outcomes between the two genotypes.

### 3.2. Overlap of DEGs

Venn diagram analysis was performed to examine the overlap of DEGs across the four comparisons, with the layout arranged in the order of basal comparison, individual infection responses, infected-genotype comparison, placing I vs. I on the far right (Figure 1B,C). For downregulated genes (Figure 1B), the *ke2*I vs. *ke2*CK comparison contained the largest number of unique genes (1243), underscoring the massive transcriptional collapse in the susceptible cultivar. Similarly, for upregulated genes (Figure 1C), *ke2*I vs. *ke2CK* dominated with 3359 unique genes. Notably, a very small core set of shared DEGs between DJCK and *ke2*CK, representing less than 5% of the total infection-responsive genes, indicating that the majority of the transcriptional reprogramming was genotype-specific.

### 3.3. GO Enrichment Analysis

Functional enrichment analysis was conducted following the logical order of CK vs. CK, each I vs. CK, and I vs. I to facilitate interpretation. In the DJCK vs. *ke2*CK comparison (Figure 1D), DEGs were significantly enriched in biological process (BP) terms associated with plant-type cell wall organization or biogenesis, sulfate transport, and lipid biosynthetic processes. The enrichment of cell wall organization terms at the basal level provided a transcriptomic rationale for the subsequent immunofluorescence analysis of pectin epitopes. In the *ke2*I vs. *ke2*CK comparison (Figure 1E), DEGs were significantly enriched in BP terms associated with the regulation of macromolecule biosynthetic processes and cellular biosynthetic processes. Prominent molecular function (MF) categories included DNA binding, transcription factor activity, and protein kinase activity, while cellular component (CC) terms were primarily associated with the extracellular region and cell wall. In the DJI vs. DJCK comparison (Figure 1F), significant enrichment was observed in the regulation of biosynthetic processes, cell wall metabolism, and primary metabolic processes, with DNA binding and transcription factor activity representing the predominant MF categories. The enrichment of cell wall metabolism genes upon infection in DJ further supported the central role of cell wall remodeling in the resistant response. Finally, in the DJI vs. *ke2*I comparison (Figure 1G), transcriptional differences between the two infected genotypes were highlighted, with enriched BP terms related to the negative regulation of catalytic activity, cell wall catabolism, pathogenesis, and response to nitrate. Enriched MF terms included heme binding and DNA binding, whereas CC categories were mainly associated with the extracellular region and apoplast. The consistent enrichment of extracellular region and cell wall-related terms across comparisons underscored the importance of cell wall dynamics in mediating the differential response to *Foc* 4.

### 3.4. KEGG Pathway Enrichment Analysis

**Figure 2:**
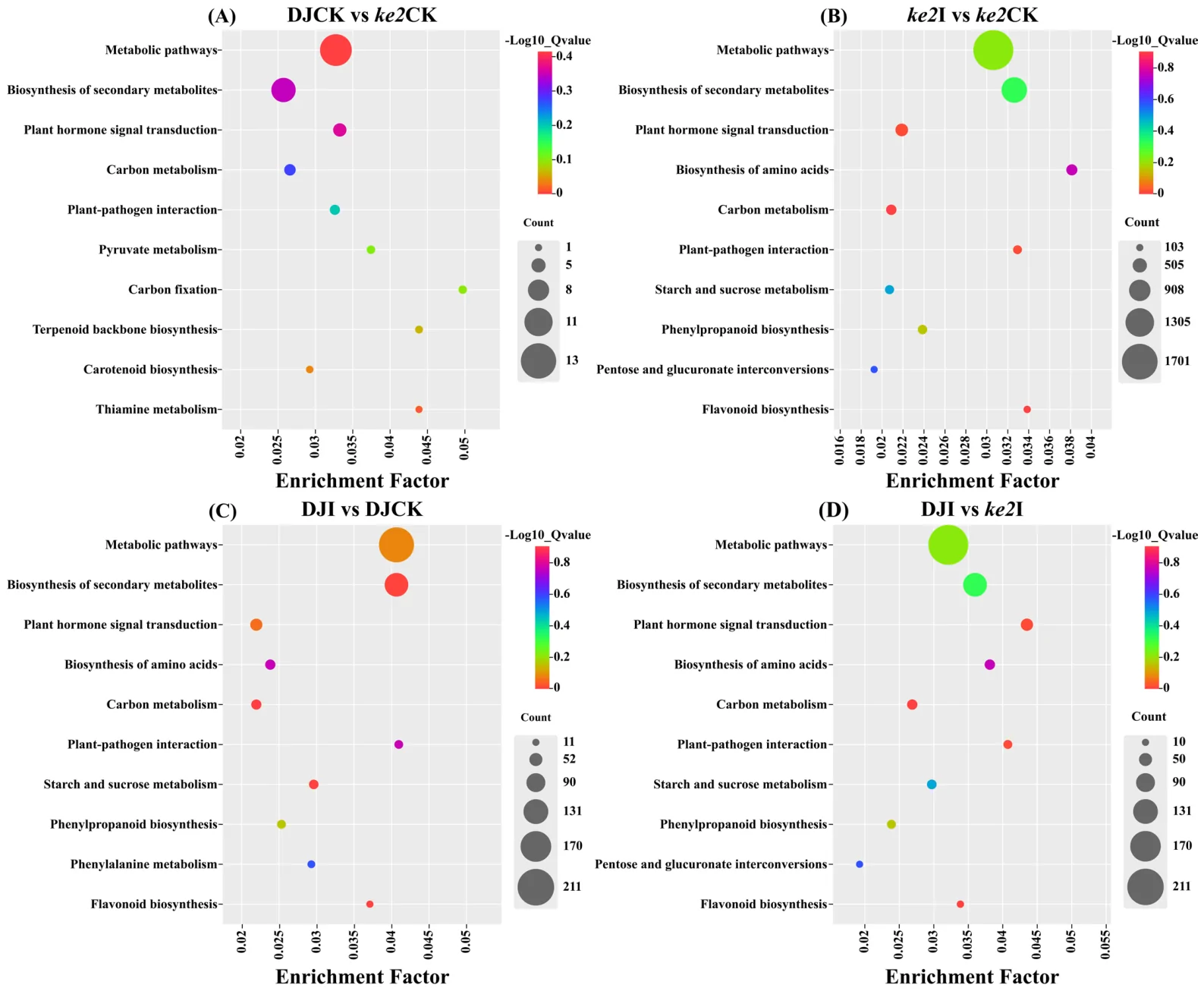
KEGG pathway enrichment analysis of differentially expressed genes. Bubble charts illustrating enriched pathways across four comparisons: (**A**) DJCK vs. *ke2*CK, (**B**) *ke2*I vs. *ke2*CK, (**C**) DJI vs. DJCK, and (**D**) DJI vs. *ke2*I. The x-axis represents the enrichment factor, the y-axis lists specific KEGG pathways, bubble size indicates the number of enriched genes (count), and color intensity indicates the significance of enrichment (−log_10_ Q-value). DJ, Dongjiao No. 1 (resistant); *ke2*, susceptible mutant; CK, control (uninoculated); I, infected with *Foc* 4.

KEGG pathway enrichment analysis was performed to identify biological pathways significantly affected across the four comparison groups, with panels arranged in the logical order of basal comparison, individual infection responses, and infected-genotype comparison (Figure 2). In the DJCK vs. *ke2*CK comparison (Figure 2A), the fewest DEGs were detected, as reflected by the smallest bubble sizes. Nevertheless, metabolic pathways exhibited the highest enrichment factor, while carbon fixation in photosynthetic organisms and thiamine metabolism also showed elevated enrichment factors, pointing to specific shifts in basal carbon processing and cofactor metabolism between the two uninfected genotypes. Biosynthesis of secondary metabolites was also represented, though with smaller gene counts. In the *ke2*I vs. *ke2*CK comparison (Figure 2B), metabolic pathways and biosynthesis of secondary metabolites remained the most consistently enriched categories, displaying the largest bubble sizes and high enrichment factors. Notably, flavonoid biosynthesis showed the highest enrichment factor among all pathways, followed by plant hormone signal transduction and carbon metabolism, indicating strong dysregulation of hormone signaling and specific secondary metabolite production in the susceptible mutant upon infection. In the DJI vs. DJCK comparison (Figure 2C), in addition to the general metabolic pathways and biosynthesis of secondary metabolites, plant–pathogen interaction and flavonoid biosynthesis exhibited high enrichment factors. Additional enrichment was observed for phenylpropanoid biosynthesis and phenylalanine metabolism, suggesting activated defense responses and secondary metabolism in the resistant cultivar following *Foc* 4 challenge. Pentose and glucuronate interconversions were also enriched, linking the transcriptomic response to cell wall polysaccharide remodeling. Finally, in the DJI vs. *ke2*I comparison (Figure 2D), metabolic pathways and biosynthesis of secondary metabolites were again prominent, while plant hormone signal transduction and flavonoid biosynthesis showed high enrichment factors. Carbohydrate metabolism pathways such as starch and sucrose metabolism and pentose and glucuronate interconversions were also enriched, underscoring the divergence in cell wall carbohydrate dynamics between the two infected genotypes. The consistent enrichment of cell wall and defense-associated pathways across the infection comparisons provided a transcriptomic basis for the subsequent enzymatic and immunolocalization analyses.

### 3.5. Expression Profiles of Defense-Related Functional Gene Modules

**Figure 3:**
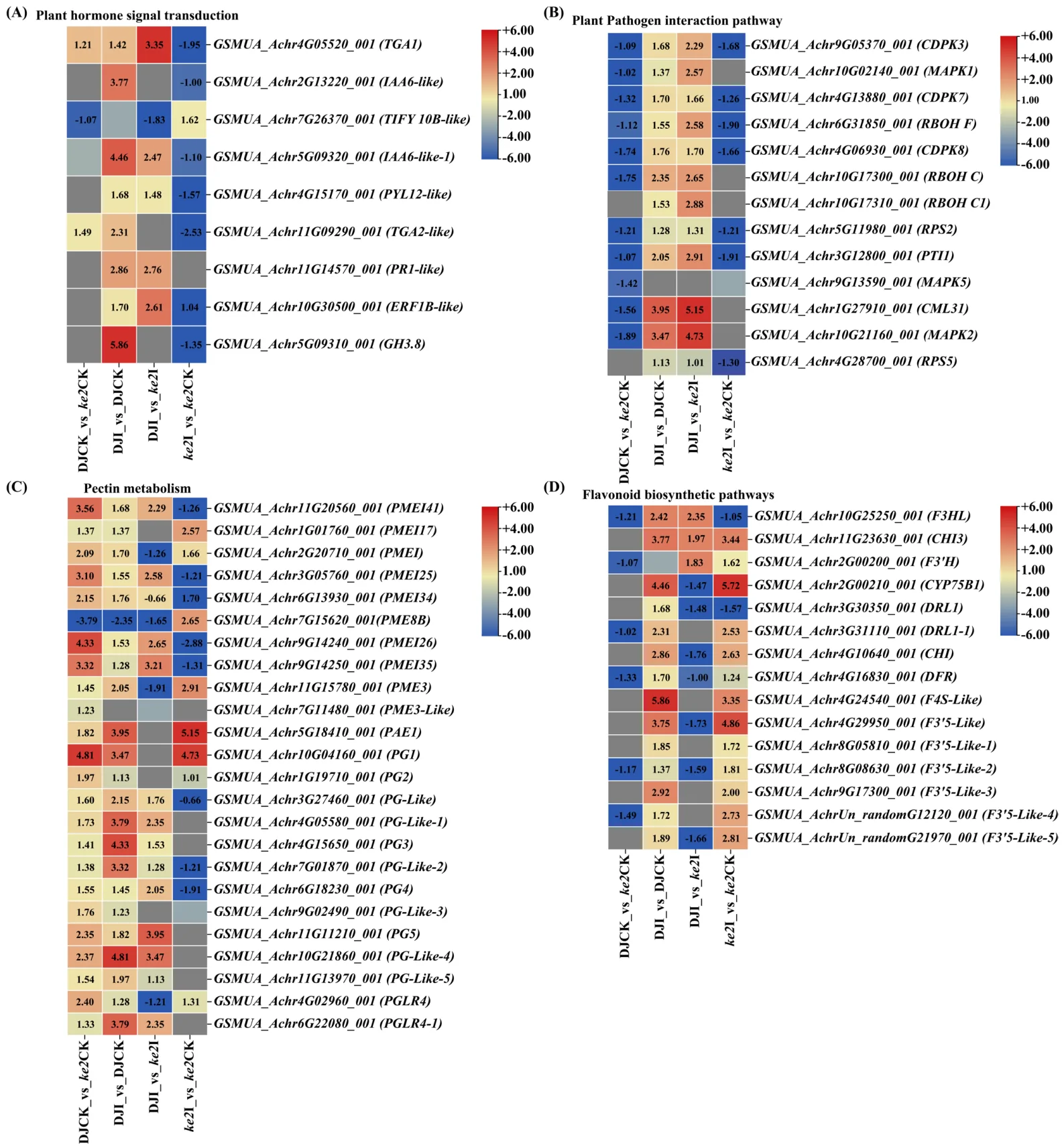
Heatmap analysis of curated defense-related genes across four comparisons. Functional modules include: (**A**) plant hormone signal transduction, (**B**) plant–pathogen interaction, (**C**) pectin metabolism, and (**D**) flavonoid biosynthesis. Columns represent comparisons in the order: DJCK_vs._*ke2*CK, *ke2*I_vs._*ke2*CK, DJI_vs._DJCK, and DJI_vs._*ke2*I. The color scale indicates expression change magnitude; red/blue represent significant differential expression (|log_2_FC| ≥ 2, FDR < 0.05), and gray indicates non-significant changes. DJ, Dongjiao No. 1 (resistant); *ke2*, susceptible mutant; CK, control (uninoculated); I, infected with *Foc* 4.

To investigate the molecular mechanisms underlying the banana response to *Foc* 4 infection, we curated a panel of defense- and cell-wall-related genes based on their significant differential expression (|log_2_FC| ≥ 2, FDR < 0.05) in at least one comparison. Their expression profiles across all four comparisons are visualized in Figure 3 to provide comparative context. The gray cells indicate non-significant changes (|log_2_FC| < 2 or FDR ≥ 0.05) in that specific pairwise comparison. The curated panel comprises genes from four functional modules including plant hormone signal transduction, plant–pathogen interaction, phenylpropanoid and flavonoid biosynthesis, and cell wall remodeling (including pectin methylesterases, polygalacturonases, and pectin acetylesterase functioning within carbohydrate and pentose/glucuronate metabolic networks).

### 3.6. Plant Hormone Signal Transduction

At the basal level (DJCK vs. *ke2*CK), minimal divergence was observed between the two cultivars, consistent with the small number of constitutive DEGs (Figure 3A). Following infection, the susceptible cultivar *ke2* exhibited broad dysregulation of hormone signaling (*ke2*I vs. *ke2*CK). For instance, the jasmonic acid repressor TIFY 10B-like was upregulated (1.62), whereas salicylic acid-responsive transcription factors TGA1 (−1.95) and TGA2-like (−2.53) were downregulated, suggesting suppressed SA-mediated defense in the susceptible genotype. By contrast, the resistant cultivar DJ mounted a more targeted hormonal response upon infection (DJI vs. DJCK). Among them, auxin-related genes IAA6-like (3.77) and IAA6-like-1 (4.46) were significantly upregulated, alongside the auxin-conjugating enzyme GH3.8 (5.86), the SA-responsive PR1-like (2.86), and the ethylene response factor ERF1B-like (1.70). In the direct comparison of infected samples (DJI vs. *ke2*I), DJ showed stronger activation of SA signaling, with TGA1 (3.35), PR1-like (2.76), and ERF1B-like (2.61) significantly upregulated, while the JA repressor TIFY10B-like (−1.83) was downregulated, indicating derepression of JA signaling in the resistant genotype.

### 3.7. Plant–Pathogen Interaction

This pathway exhibited the most dramatic infection-induced changes in DJ (Figure 3B). At the basal level (DJCK vs. *ke2*CK), most pattern-triggered immunity (PTI) genes showed no significant difference between the two uninfected cultivars. Upon infection, *ke2* displayed general downregulation of defense signaling genes (*ke2*I vs. *ke2*CK), including *MAPK2*, *CDPK8*, and members of the RBOH family. Conversely, DJ specifically upregulated core PTI components (DJI vs. DJCK). The *CML31* (3.95), *MAPK2* (3.47), *PTI1*, and *RBOH C* (2.35). The direct comparison of infected tissues (DJI vs. *ke2*I) revealed near-universal upregulation of these defense genes in DJ, with notably high expression of *CML31* (5.15), *MAPK2* (4.73), *PTI1* (2.91), *RBOH C1* (2.88), and *RBOH C* (2.65); calcium-dependent protein kinases (*CDPK3*, *CDPK7*, *CDPK8*) and *MAPK1* were also upregulated in DJI relative to *ke2*I. These data indicate that DJ activates a robust Ca^2+^/MAPK/ROS signaling hub upon infection that remains largely dormant in *ke2*.

### 3.8. Pectin Metabolism

Consistent with the minimal basal transcriptional divergence, most curated pectin-modifying genes showed non-significant differences between the two uninfected cultivars (DJCK vs. *ke2*CK; gray cells in Figure 3C). Nevertheless, several transcripts trended toward higher basal abundance in DJ, including pectin methylesterase inhibitors (*PMEI26*, 4.33; *PMEI41*, 3.56; *PMEI25*, 3.10), polygalacturonases (*PG1*, 4.81; *PG-Like-4*, 2.37), and *PAE1* (1.82), whereas *PME8B* (−3.79) trended lower. Following infection, DJ displayed widespread upregulation of pectin-degrading enzyme genes (DJI vs. DJCK), with strong induction of *PME3-Like* (3.95), *PAE1* (3.95), *PG3* (4.33), *PG-Like-4* (4.81), and several PMEIs (*PMEI26*, 1.53; *PMEI41*, 1.68). In contrast, the susceptible cultivar *ke2* exhibited a divergent response (*ke2*I vs. *ke2*CK). Most PMEIs were downregulated (*PMEI26*, −2.88; *PMEI41*, −1.26), while *PME8B* (2.65), *PME3* (2.91), and *PAE1* (5.15) were upregulated. Direct comparison of infected samples (DJI vs. *ke2*I) revealed clear divergence. DJ showed higher expression of multiple PME inhibitors (*PMEI35*, 3.21; *PMEI26*, 2.65) and polygalacturonases (*PG-Like-4*, 3.47; *PG5*, 3.95), whereas *ke2* showed higher expression of *PME8B* (−1.65), *PME3* (−1.91), and *PME1* (−1.26). Notably, the resistant cultivar specifically induced the *PME3-Like* isoform upon infection, whereas the sensitive cultivar upregulated the distinct *PME3* isoform, indicating isoform-specific regulatory control of pectin degradation during defense activation.

### 3.9. Flavonoid Biosynthesis

Heatmap analysis revealed distinct expression patterns across the four comparisons (Figure 3D). At the basal level (DJCK vs. *ke2*CK), most flavonoid genes showed no significant difference, though several (*DFR*, −1.33; *F3′5′-Like-4*, −1.49) trended toward lower expression in DJ, indicating marginally higher basal levels in *ke2* for those transcripts. Following infection, the sensitive cultivar *ke2* showed stronger transcriptional activation (*ke2*I vs. *ke2*CK) than the resistant DJ. The key genes, including *CYP75B1* (5.72), *F3′5′-Like* (4.86), and *CHI3* (3.44), were strongly upregulated, while *DRL1* (−1.57) and *F3HL* (−1.05) were downregulated. DJ also upregulated flavonoid genes upon infection (DJI vs. DJCK), notably *F4S-Like* (5.86), *CYP75B1* (4.46), and *CHI3* (3.77). In the direct comparison of infected samples (DJI vs. *ke2*I), most genes showed higher expression in *ke*2 (*CYP75B1*, −1.47; *CHI*, −1.76), except *F3HL* (2.35), *CHI3* (1.97), and *F3′H* (1.83), which were significantly higher in DJI. These results suggest that while *ke2* mounts a broad but potentially dysregulated flavonoid response, DJ selectively upregulates specific branches of the phenylpropanoid pathway.

### 3.10. Enzyme Activity Analyses of PME and PG

To functionally validate the transcriptomic patterns of pectin remodeling, PME and PG activities were quantified in root and leaf tissues. Root enzyme activities were assessed across all three developmental stages, whereas leaf enzyme activities were analyzed at the TC and BSS stages.

**Figure 4:**
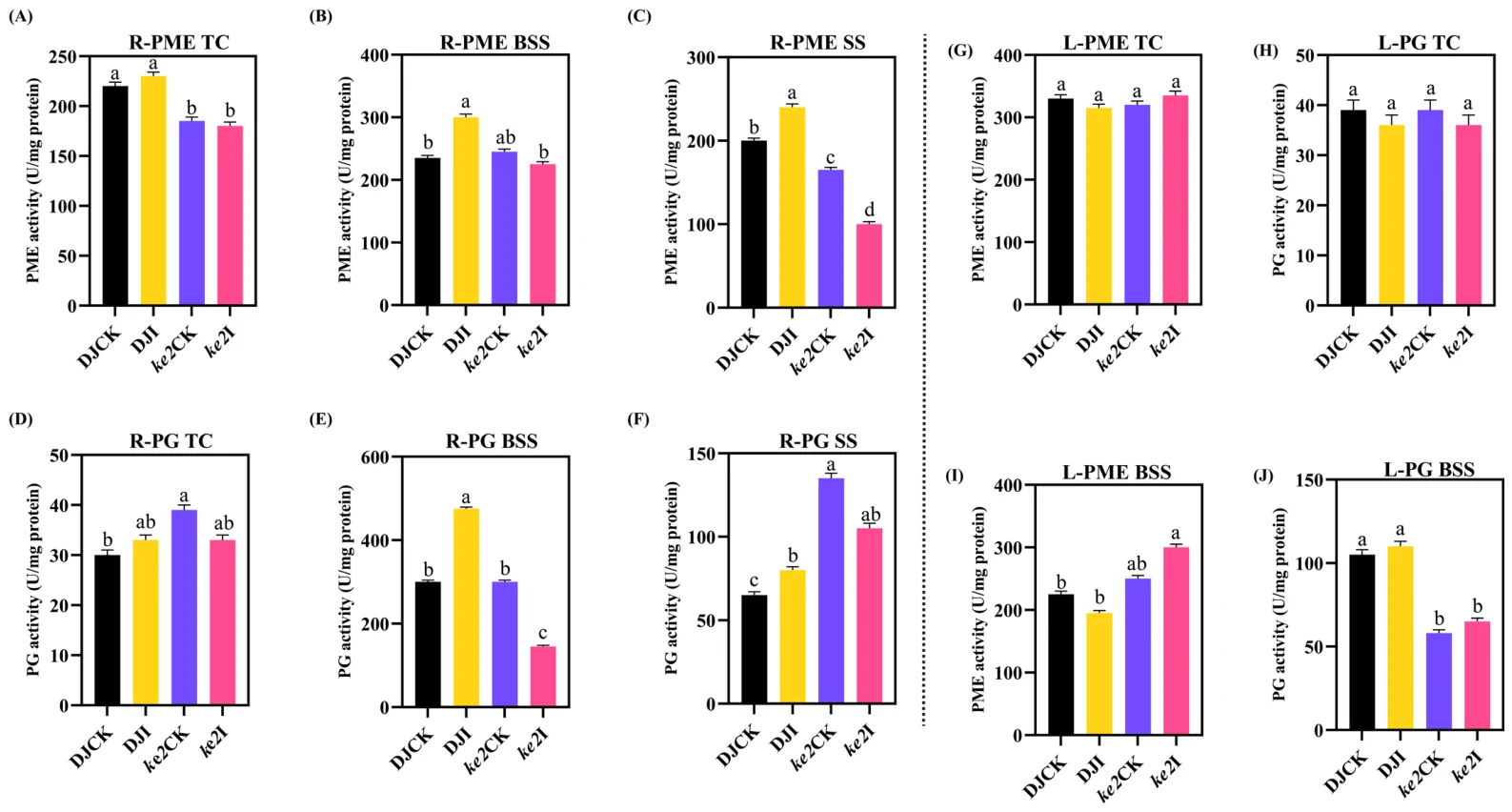
Enzymatic activity of pectin methylesterase (PME) and polygalacturonase (PG). (**A**–**C**) Root PME (R-PME) activity at tissue culture (TC), bag seedling stage (BSS), and shooting stage (SS). (**D**–**F**) Root PG (R-PG) activity at TC, BSS, and SS. (**G**,**H**) Leaf PME (L-PME) activity at TC and BSS. (**I**,**J**) Leaf PG (L-PG) activity at TC and BSS. Data are presented as mean ± standard deviation. Different lowercase letters (a–d) above bars denote statistically significant differences among sample groups within each panel (*p* < 0.05). DJ, Dongjiao No. 1 (resistant); *ke2*, susceptible mutant; CK, control (uninoculated); I, infected with *Foc* 4.

Root PME (R-PME) activity varied significantly among samples and developmental stages (Figure 4A–C). At the TC stage, DJI exhibited the highest activity (231.45 U/mg). The resistant cultivar showed significantly higher PME activity than the susceptible mutant both before and after pathogen infection, though infection did not cause significant changes in either genotype. At the BSS stage, DJI maintained the highest activity (300 U/mg), significantly exceeding DJCK and *ke2*I. An increase in PME activity was only observed in the resistant cultivar. A similar result was observed at the SS stage. DJI again showed the highest activity (250 U/mg), while *ke2*I displayed the lowest activity (100 U/mg), indicating a progressive decline in R-PME activity in the susceptible genotype over time.

Root PG (R-PG) activity showed infection-response trends similar to those of R-PME. Pathogen infection increased activity in DJ while decreasing it in *ke2* (Figure 4D–F). However, the susceptible mutant generally exhibited higher basal PG activity. At the TC stage, *ke2*CK exhibited the highest activity (39 U/mg), significantly surpassing DJCK. At the BSS stage, DJI showed the highest R-PG activity (480 U/mg), markedly exceeding all other samples, whereas *ke2*I showed the lowest activity (140 U/mg). At the SS stage, *ke2*CK again displayed the highest activity (135 U/mg), significantly higher than DJCK.

In leaf tissues, neither PME nor PG activities differed significantly among the four sample groups at the TC stage (Figure 4G–J), suggesting that the short infection duration was insufficient for systemic defense signals to translocate to leaves. At the BSS stage, pathogen infection did not significantly alter either enzyme activity. However, L-PME activity was higher in the susceptible mutant than in the resistant cultivar, whereas L-PG activity showed the opposite pattern: DJI exhibited the highest L-PG activity (115 U/mg), while *ke2*CK and *ke2*I showed significantly lower activity.

Collectively, these results indicate differential spatiotemporal regulation of pectin-degrading enzymes between the resistant and susceptible genotypes. DJI generally maintained higher root enzyme activity across developmental stages, particularly at the BSS stage, a critical window for *Foc* 4 infection, whereas *ke2*I showed stagnant or declining activity. The contrasting leaf patterns, in which *ke2*I exhibited elevated L-PME activity at the BSS stage while DJI showed elevated L-PG activity, suggest tissue-specific roles for pectin remodeling during defense.

### 3.11. Immunolocalization of PME-Associated Epitopes in Banana Root and Leaf

To visualize the spatial distribution and abundance of PME-related epitopes, root and leaf cross-sections from the resistant cultivar DJ and *ke2* were examined by immunofluorescence labeling at the TC, BSS, and SS in roots, and in leaves.

**Figure 5:**
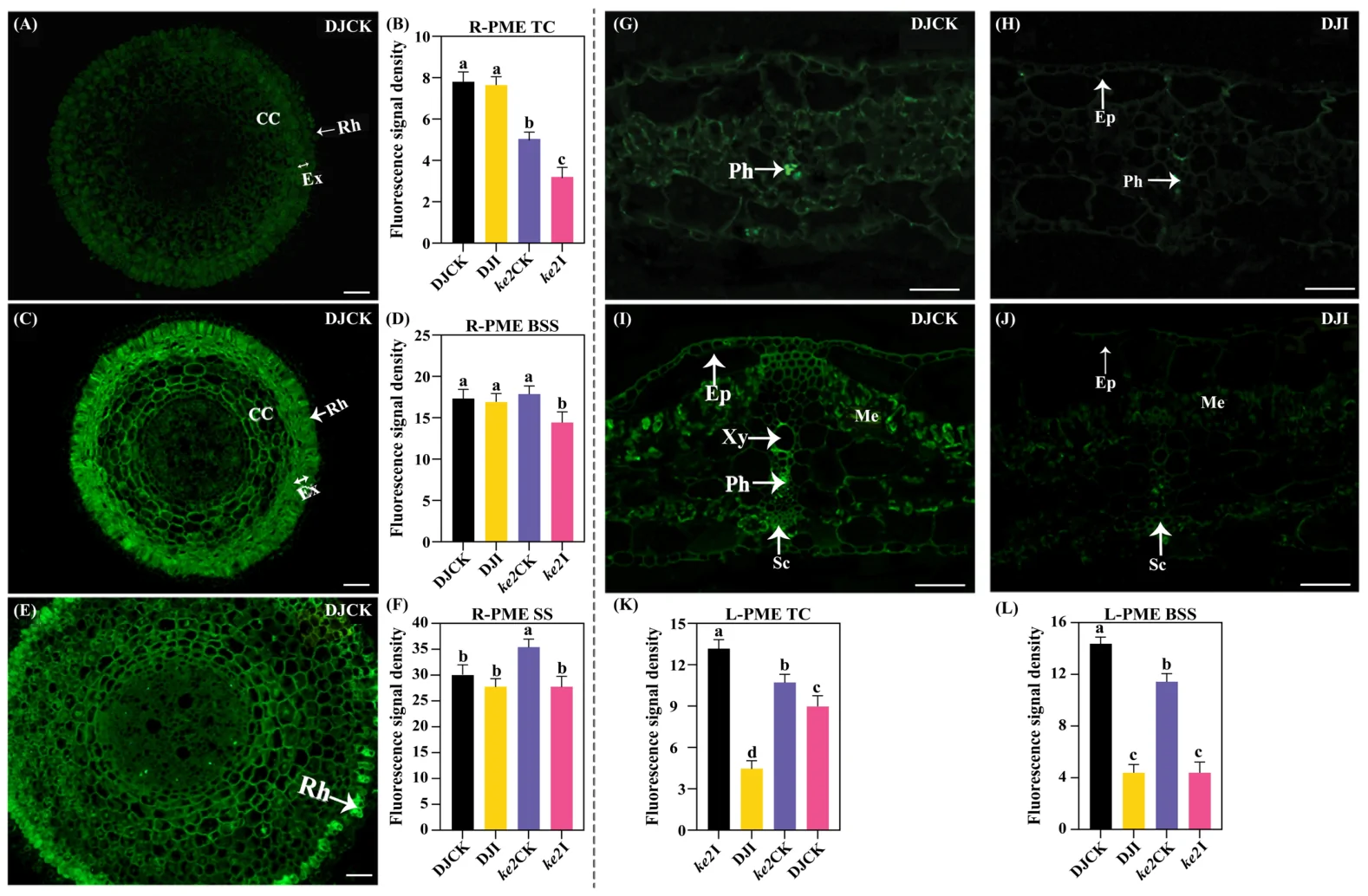
Immunolocalization and quantitative analysis of pectin methylesterase (PME) in banana (*Musa spp*. AAA) root and leaf tissues. Fluorescence microscopy images showing PME distribution in root and leaf cross-sections at tissue culture (TC), bag seedling stage (BSS), and shooting stage (SS). (**A**,**C**,**E**) Representative images of the resistant cultivar Dongjiao No. 1 control (DJCK) root cross-sections at TC, BSS, and SS, respectively. (**B**,**D**,**F**) Quantification of root PME (R-PME) fluorescence signal density across the four sample groups at TC, BSS, and SS, respectively. (**G**,**I**) Representative images of DJCK leaf cross-sections at TC and BSS. (**H**,**J**) Representative images of DJ infected with *Foc* 4 (DJI) leaf cross-sections at TC and BSS. (**K**,**L**) Quantification of leaf PME (L-PME) fluorescence signal density at TC and BSS, respectively. Green fluorescence indicates PME immunolocalization. Anatomical structures are labeled: CC, cortical cells; Rh, rhizodermis; Ex, exodermis; Ep, epidermis; Me, mesophyll; Xy, xylem; Ph, phloem; Sc, sclerenchyma. Scale bars = 50 μm. Data are presented as mean ± standard deviation. Different lowercase letters above bars indicate statistically significant differences among sample groups (*p* < 0.05). DJ, Dongjiao No. 1 (resistant); *ke2*, susceptible mutant; CK, control (uninoculated); I, infected with *Foc* 4.

At the TC stage, strong green fluorescence was detected in the CC, Rh, and Ex of DJ roots (Figure 5A). Quantitative analysis revealed that both DJCK and DJI maintained the highest and comparable fluorescence signal densities, whereas *ke2*CK showed a significantly lower level, and *ke2*I exhibited the lowest density among all groups (Figure 5B). At the BSS stage, fluorescence was more broadly distributed throughout the root cross-sections in DJCK (Figure 5C). Signal quantification indicated that DJCK, DJI, and *ke2*CK shared similarly high fluorescence densities, while *ke2*I displayed a marked and significant reduction (Figure 5D). At the SS stage, fluorescence remained prominent in the Rh of DJCK (Figure 5E). Notably, *ke2*CK showed the highest fluorescence density at this stage, whereas DJCK, DJI, and *ke2*I were statistically similar at lower levels (Figure 5F). These results indicate that the resistant genotype DJ maintains stable, high levels of PME-associated epitopes in roots upon infection during early and middle developmental stages, whereas the susceptible mutant *ke2* fails to sustain these levels following *Foc* 4 challenge.

In leaf cross-sections at the TC stage, fluorescence was primarily localized in the Ph of DJCK (Figure 5G), whereas DJI showed a dramatically weaker signal (Figure 5H). Quantification confirmed that DJCK possessed the highest fluorescence density, followed by *ke2*CK and *ke2*I at intermediate levels, while DJI displayed the lowest density (Figure 5K). At the BSS stage, fluorescence in DJCK leaves was distributed across the Ep, Me, Xy, Ph, and Sc (Figure 5I), but was substantially diminished in DJI (Figure 5J). Signal density analysis showed that DJCK maintained the highest level, *ke2*CK showed an intermediate level, and both DJI and *ke2*I were significantly reduced to comparably low levels (Figure 5L). Collectively, these data demonstrate that DJ leaves harbor high constitutive levels of PME-associated epitopes that are strongly attenuated by *Foc* 4 infection, whereas *ke2* leaves exhibit moderate basal levels that are also suppressed upon infection at the BSS stage.

### 3.12. Immunolocalization of Low Methyl-Esterified Pectin in Root and Leaf (JIM5)

**Figure 6:**
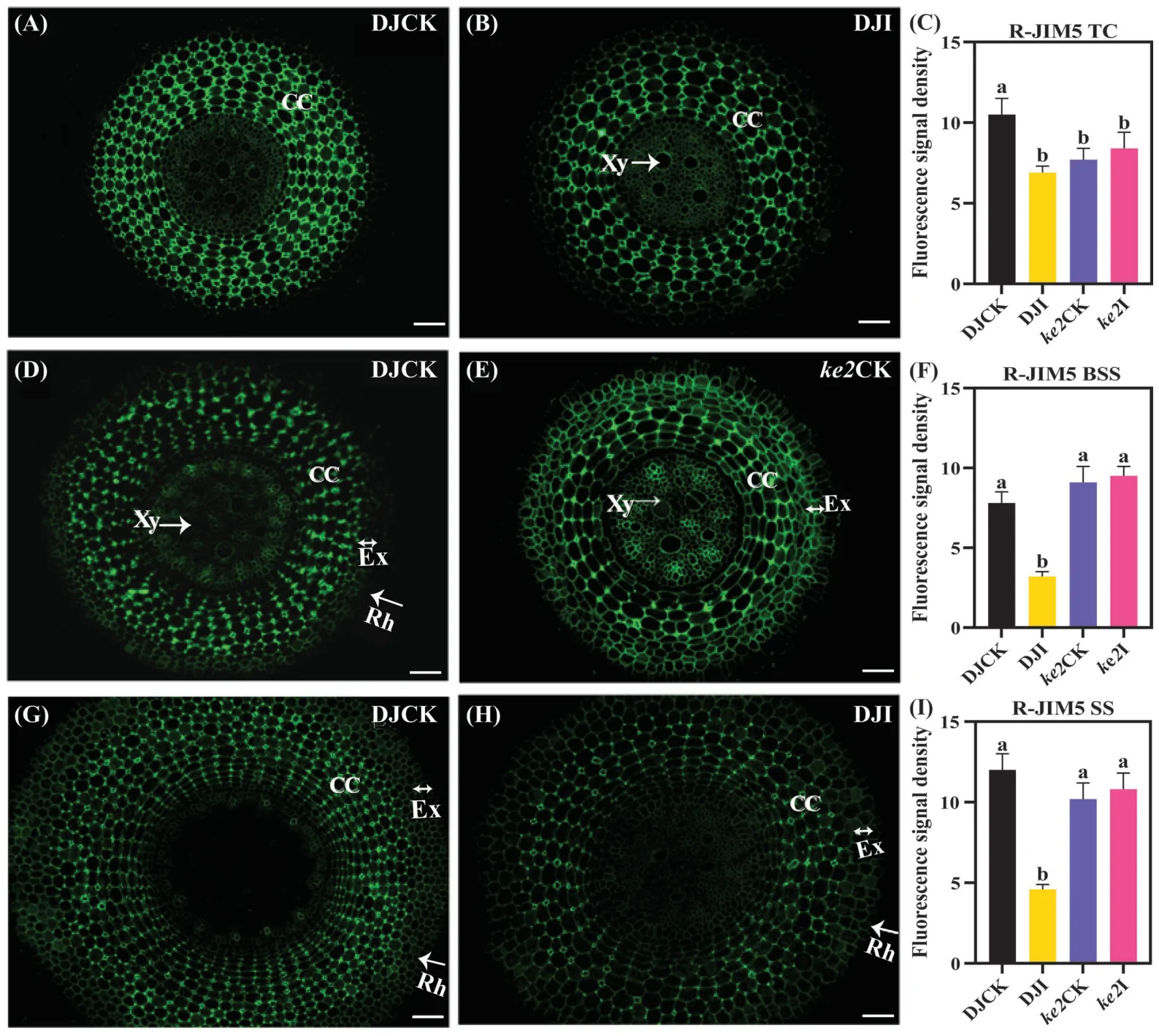
Immunolocalization and quantitative analysis of low methyl-esterified pectin in banana (*Musa* spp. AAA) root tissues using JIM5 antibody. Fluorescence microscopy images and quantification of JIM5 epitope distribution in root cross-sections at tissue culture (TC), bag seedling stage (BSS), and shooting stage (SS). Anatomical structures are labeled: CC, cortical cells; Xy, xylem; Ex, exodermis; En, endodermis. Scale bars = 50 μm. (**A**,**B**) Representative images of DJCK and DJI at TC. (**D**,**E**) Representative images of DJCK and *ke2*CK at BSS. (**G**,**H**) Representative images of DJCK and DJI at SS. (**C**,**F**,**I**) Quantification of JIM5 fluorescence signal density across the four sample groups at TC, BSS, and SS, respectively. Data are presented as mean ± standard deviation. Different lowercase letters above bars indicate statistically significant differences (*p* < 0.05). DJ, Dongjiao No. 1 (resistant); *ke2*, susceptible mutant; CK, control (uninoculated); I, infected with *Foc* 4.

The distribution and abundance of low methyl-esterified pectin in root tissues were analyzed using the JIM5 antibody across banana root cross-sections at three developmental stages (Figure 6A–I). The green fluorescence signal indicates the presence of the JIM5 epitope, primarily localized in the cell walls of cortical cells, with additional signals in the xylem, exodermis, rhizodermis, and phloem cells at the BSS stage. Quantitative analysis of fluorescence signal density at TC stage revealed that DJCK consistently exhibited the highest signal density across all three stages. *Foc* 4 infection significantly decreased the epitope content only in the resistant cultivar, whereas the other three sample groups (DJI, *ke2*CK, and *ke2*I) showed no significant differences among themselves (Figure 6A–C). At both BSS and SS stages, DJI displayed the lowest signal intensity while there were no differences among the other three samples (Figure 6D–I). This reduction in root cell wall pectin methylesterification in DJI suggests active pectin de-esterification or depolymerization in the resistant cultivar following *Foc* 4 infection, aligning with the transcriptomic enrichment of cell wall metabolism and extracellular region terms observed in the GO and KEGG analyses.

**Figure 7:**
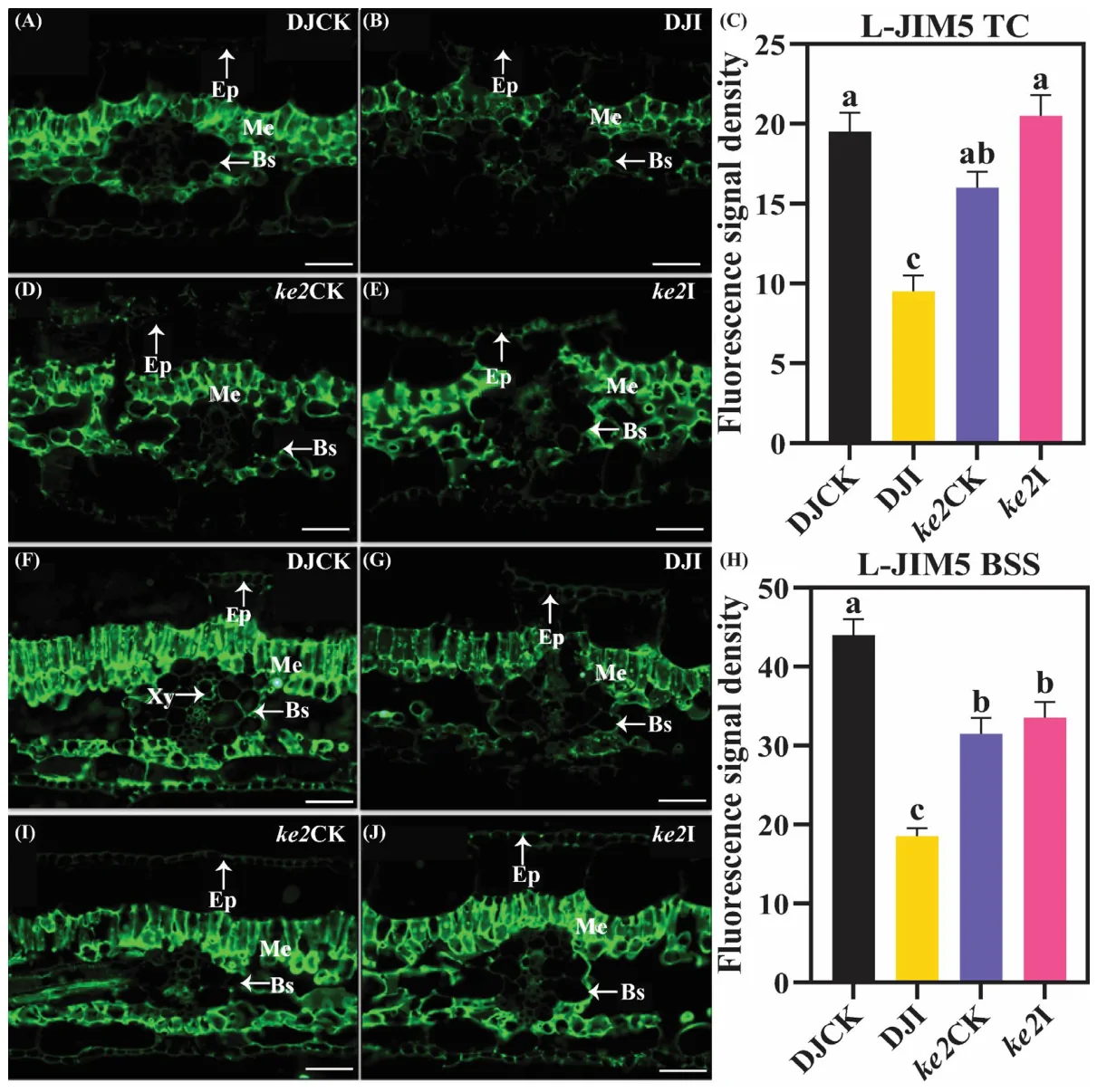
Immunolocalization and quantitative analysis of low methyl-esterified pectin in banana (*Musa* spp. AAA) leaf tissues using JIM5 antibody. Fluorescence microscopy images showing JIM5 epitope distribution in leaf cross-sections at tissue culture (TC) and bag seedling stage (BSS). Anatomical structures are labeled: Ep, epidermis; Me, mesophyll; BS, bundle sheath; Xy, xylem. Scale bars = 50 μm. (**A**,**B**) Representative images of DJCK and DJI at TC. (**D**,**E**) Representative images of *ke2*CK and *ke2*I at TC. (**F**,**G**) Representative images of DJCK and DJI at BSS. (**I**,**J**) Representative images of *ke2*CK and *ke2*I at BSS. (**C**) Quantification of JIM5 fluorescence signal density at TC. (**H**) Quantification at BSS. Data are presented as mean ± standard deviation. Different lowercase letters above bars indicate statistically significant differences (*p* < 0.05). DJ, Dongjiao No. 1 (resistant); *ke2*, susceptible mutant; CK, control (uninoculated); I, infected with *Foc* 4.

The distribution and abundance of low methyl-esterified pectin in leaf tissues were analyzed using the JIM5 antibody at the TC and BSS stages (Figure 7). The green fluorescence signal indicates the presence of the epitope, mainly localized in the cell walls of mesophyll cells and the epidermis at BSS and SS stages. At the TC stage (Figure 7A–E), the DJI group exhibited the lowest fluorescence signal density, significantly lower than DJCK and *ke2*I, while *ke2*CK showed an intermediate level (Figure 7C). At the BSS stage (Figure 7F–J), DJCK displayed the highest fluorescence signal density, whereas DJI showed a significantly reduced signal, lower than all other groups. The *ke2*CK and *ke2*I groups showed intermediate signal densities, significantly higher than DJI but lower than DJCK (Figure 7H). Across both stages, DJI consistently demonstrated the lowest levels of JIM5-recognized pectin epitopes in leaf tissue. This reduction in DJI compared to the controls (DJCK, *ke2*CK) and *ke2*I parallels the reduction observed in root tissue and supports the conclusion that *Foc* 4 infection triggers active pectin de-esterification in the resistant cultivar DJ.

### 3.13. Immunolocalization of Highly Methyl-Esterified Pectin (JIM7)

**Figure 8:**
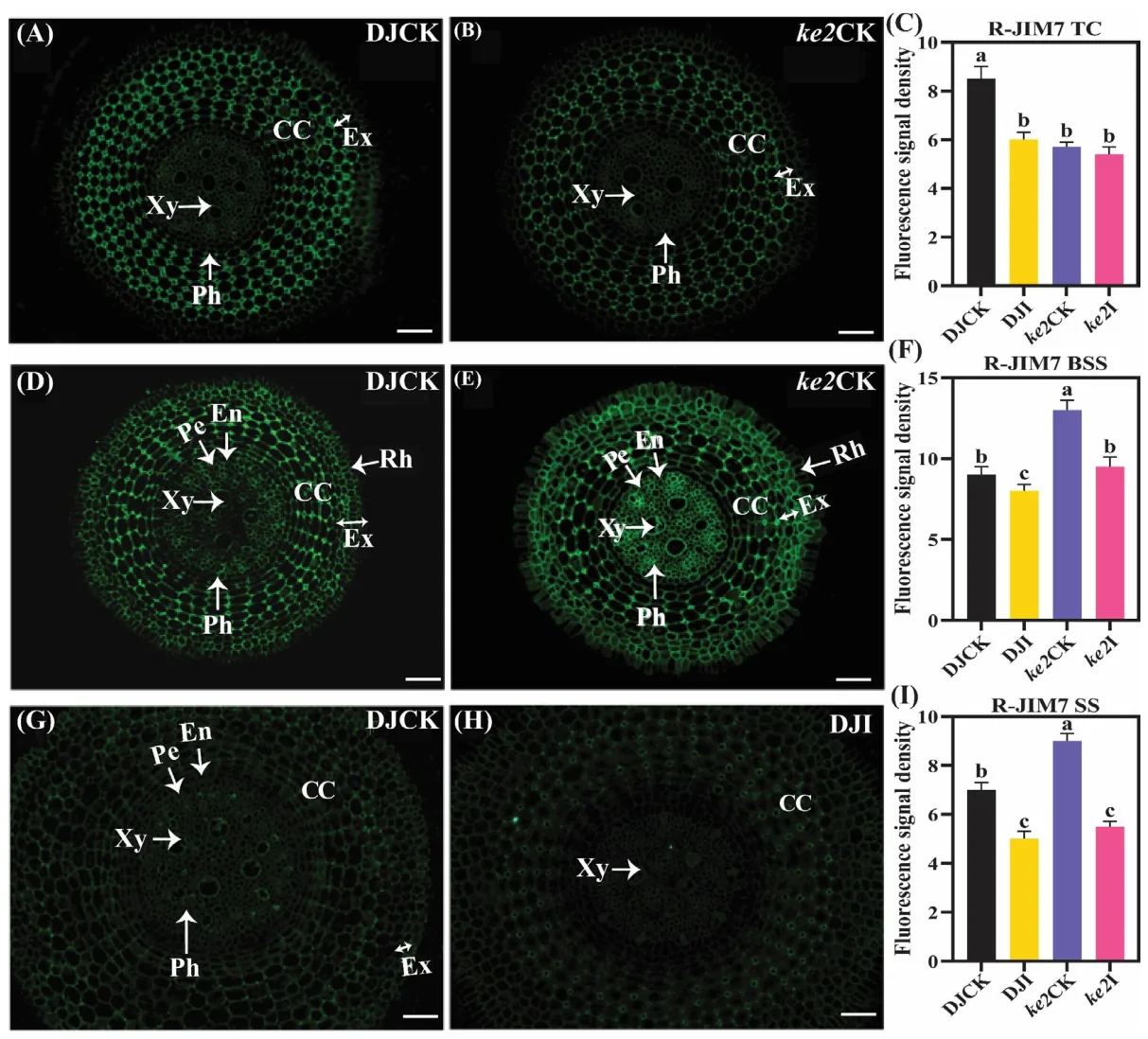
Immunolocalization and quantitative analysis of highly methyl-esterified pectin in banana (*Musa* spp. AAA) root tissues using JIM7 antibody. Fluorescence microscopy images showing JIM7 epitope distribution in root cross-sections at tissue culture (TC), bag seedling stage (BSS), and shooting stage (SS). Anatomical structures are labeled: CC, cortical cells; Xy, xylem; Ph, phloem; Ex, exodermis; En, endodermis; Pe, pericycle; Rh, rhizodermis. Scale bars = 50 μm. (**A**,**B**) Representative images of DJCK and DJI at TC. (**D**,**E**) Representative images of DJCK and *ke2*CK at BSS. (**G**,**H**) Representative images of DJCK and DJI at SS. (**C**,**F**,**I**) Quantification of JIM7 fluorescence signal density across the four sample groups at TC, BSS, and SS, respectively. Data are presented as mean ± standard deviation. Different lowercase letters above bars indicate statistically significant differences (*p* < 0.05). DJ, Dongjiao No. 1 (resistant); *ke2*, susceptible mutant; CK, control (uninoculated); I, infected with *Foc* 4.

The distribution and abundance of highly methyl-esterified (40–80%) pectin in root tissues were analyzed using the JIM7 antibody across banana root cross-sections at three developmental stages, including TC, BSS, and SS (Figure 8). The green fluorescence signal indicates the presence of the JIM7 epitope, primarily localized in the cell walls of cortical cells and vascular tissues. At the TC stage (Figure 8A–C), DJCK exhibited significantly higher fluorescence signal density than *ke2*CK, but this was not the case after pathogen infection, because *Foc* 4 infection decreased the epitope content only in the resistant cultivar. At the BSS stage (Figure 8D–F), *ke2*CK showed the highest signal density, significantly higher than all other groups. Pathogen infection caused a decrease in the epitope content in both types. The susceptible mutant still displayed higher signal intensity than DJ after *Foc* 4 infection. At the SS stage (Figure 8G–I), the signal intensity was markedly weaker than at the earlier stages, with a labeling pattern similar to that at the BSS stage, except that the epitope content in both genotypes was at the same level after pathogen infection.

**Figure 9:**
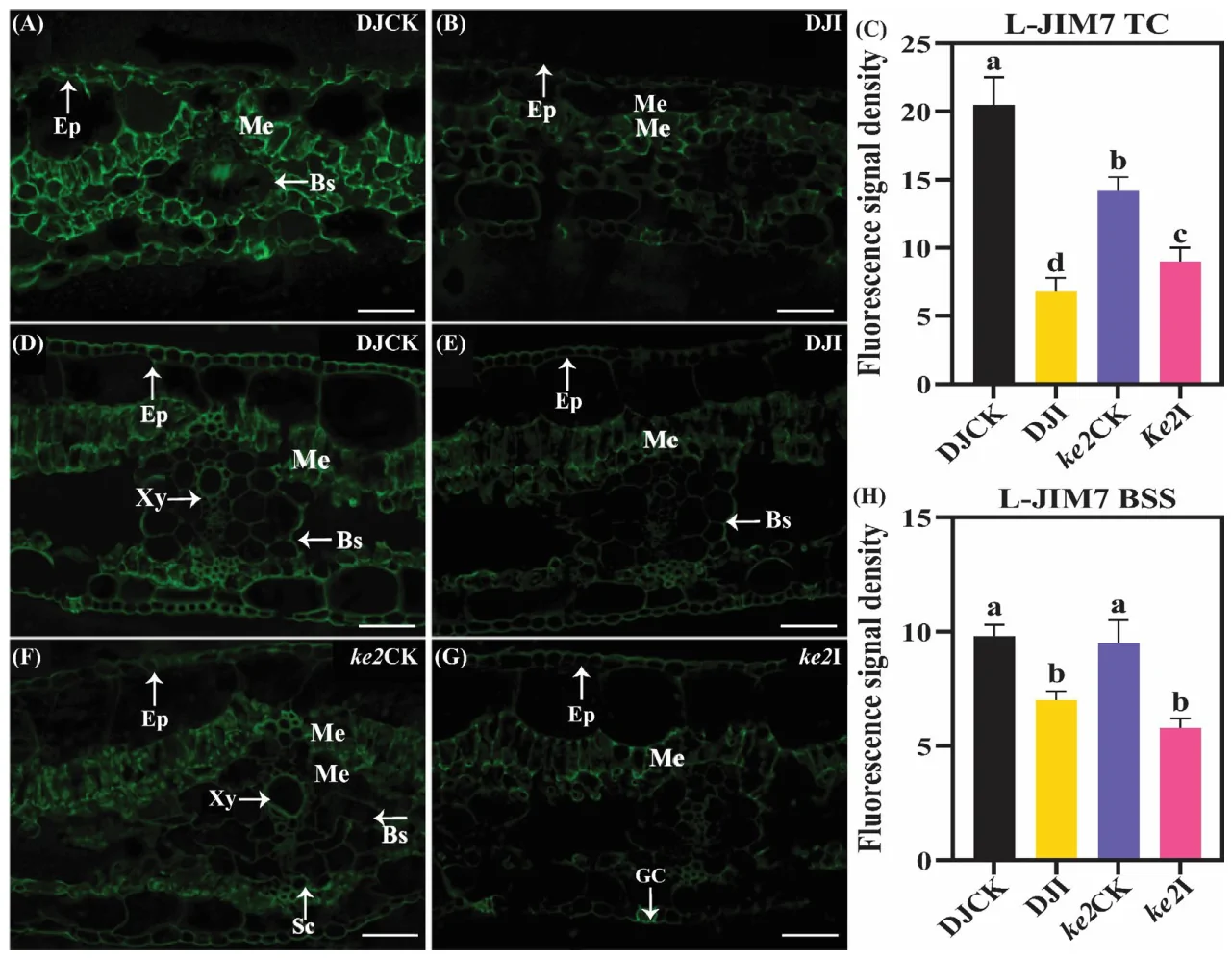
Immunolocalization and quantitative analysis of highly methyl-esterified pectin in banana (*Musa* spp. AAA) leaf tissues using JIM7 antibody. Fluorescence microscopy images showing JIM7 epitope distribution in leaf cross-sections at tissue culture (TC) and bag seedling stage (BSS). Anatomical structures are labeled: Ep, epidermis; Me, mesophyll; BS, bundle sheath; Xy, xylem; Sc, sclerenchyma; GC, guard cell. Scale bars = 50 μm. (**A**,**B**) Representative images of DJCK and DJI at TC. (**D**,**E**) Representative images of DJCK and DJI at BSS. (**F**,**G**) Representative images of *ke2*CK and *ke2*I at BSS. (**C**) Quantification of JIM7 fluorescence signal density at TC. (**H**) Quantification at BSS. Data are presented as mean ± standard deviation. Different lowercase letters above bars indicate statistically significant differences (*p* < 0.05). DJ, Dongjiao No. 1 (resistant); *ke2*, susceptible mutant; CK, control (uninoculated); I, infected with *Foc* 4.

The distribution and abundance of highly methyl-esterified pectin in leaf tissues were analyzed using the JIM7 antibody at the TC and BSS stages (Figure 9). The green fluorescence signal indicates the presence of the epitope, distributed across the leaf section except the bundle sheath, primarily localized in the cell walls of the epidermis, mesophyll, and the xylem. At the TC stage (Figure 9A–C), both the resistant cultivar and the susceptible mutant exhibited decreased epitope content upon *Foc* 4 infection. DJCK displayed the highest signal density, significantly higher than *ke2*CK. After infection, the JIM7 epitope decreased in both genotypes, with DJI showing significantly lower levels than *ke*2I.

## 4. Discussion

Fusarium wilt is a devastating disease of banana, a major fruit crop cultivated throughout tropical and subtropical regions. The interaction between banana and *Foc* 4 represents one of the most devastating plant–pathogen confrontations in global agriculture. Due to the pathogen’s high ability to spread and its exceptional persistence in the environment, it inflicts severe, widespread damage on banana production around the world [4]. Acting as the initial line of defense, the PCW is crucial in modulating subsequent plant immune responses. Its biogenesis is governed by multiple regulatory mechanisms, particularly those involving phytohormone biosynthesis and signaling [4], plant pathogen interaction pathways [30,31], flavonoid biosynthesis [32], and the obvious PME metabolism genes [16]. In this study, we present an in-depth examination of the physiological, cellular, and transcriptomic changes occurring in the banana cultivar DJ and its susceptible mutant *ke2*, following exposure to the *Foc* 4 pathogen. Our transcriptomic analysis revealed a striking paradox. The susceptible cultivar *ke2* mounted a vastly larger transcriptional response to *Foc* 4 infection (4493 DEGs) compared to the resistant cultivar DJ (430 DEGs). This pattern aligns with emerging evidence that susceptibility is often associated with dysregulated, hyperactive transcriptional reprogramming rather than a mere absence of defense responses [33]. Furthermore, the minimal transcriptional differences observed between DJCK and *ke2*CK (15 DEGs; Figure 1A) suggest that resistance in DJ is not primarily based on constitutive defense activation but instead relies on the infection-induced, coordinated defense cascade following pathogen recognition.

### 4.1. Plant Cell Wall Integrity and Modification Drive Banana Defense Against Foc 4

RNA-seq-based transcriptomic profiling offers valuable insights into the complex defense responses of plants under biotic stress [34,35]. In this study, we detected a large number of DEGs in DJ and *ke*2 following *Foc* 4 inoculation. GO (Figure 1D–G) and KEGG (Figure 2) enrichment analyses consistently identified cell wall-related processes as a nexus of differential regulation between DJ and *ke*2. The resistant cultivar exhibited basal enrichment for plant-type cell wall organization (DJCK vs. *ke*2CK) and specific upregulation of cell wall metabolism upon infection (DJI vs. DJCK), whereas *ke*2 showed broader but less focused enrichment in general biosynthetic processes. This finding resonates with the established concept that cell walls serve as the first line of defense against vascular wilt pathogens [4]. Additionally, Venn diagram analysis revealed a core set of commonly upregulated and downregulated DEGs shared between DJ and *ke*2 both before and after infection, indicating a conserved basal transcriptional response to the pathogen (Figure 1B,C).

Transcriptomic analysis of pectin metabolism genes revealed a striking difference between DJ and *ke2*. Upon *Foc* 4 infection, DJ massively upregulated key pectin-degrading enzyme genes, including *PME3-Like*, *PAE1*, and multiple polygalacturonases (*PG1*, *PG3*, *PG5*, *PG-Like-4*) (Figure 3). This coordinated upregulation of PME, PAE, and PG constitutes a complete pectin degradation cascade. PME de-esterifies homogalacturonan, PAE removes acetyl groups to further expose the backbone, and PG cleaves the de-esterified pectin into oligogalacturonides (OGs) [36]. In contrast, *ke2* failed to activate this enzymatic machinery upon infection (Figure 3). Notably, while DJ also upregulated several PMEIs, the dominant defense-related isoform *PME3-Like* escaped inhibition (Figure 3), suggesting isoform-specific regulatory control that permits targeted pectin degradation while limiting excessive damage [12].

Performing PME and PG enzymatic activity assays provides functional validation for these transcriptional patterns [22]. DJI consistently exhibited higher root PME and PG activities across developmental stages, with the most pronounced increase observed at the bag seedling stage (BSS; Figure 4), a critical vulnerability window for *Foc* 4 infection. Conversely, *ke2*I showed declining or stagnant enzyme activities, particularly at the shooting stage (SS; Figure 4). This developmental timing suggests that DJ deploys pectin degradation-mediated immunity precisely when the pathogen is most likely to establish infection. The positive correlation between induced PME activity and disease resistance observed here is consistent with recent functional evidence in grapevine, in which knockout of *PME10* abolished infection-induced PME activity and increased susceptibility to *B. cinerea*, whereas overexpression enhanced resistance [37]. Collectively, these cross-species findings support a model in which infection-induced PME activity is not merely a passive consequence of infection but an active, genetically controlled defense component.

To further delineate the spatial basis of these enzymatic differences, immunolocalization of PME protein was performed in root and leaf tissues (Figure 5). In roots, DJ exhibited high constitutive PME levels that remained stable upon infection at the tissue culture and bag seedling stages, whereas the susceptible mutant *ke2* displayed infection-induced depletion of PME epitopes and failed to sustain abundance at the shooting stage (Figure 5A–F). In leaves, DJ maintained the highest basal PME signal density, which was strongly attenuated by *Foc* 4 infection; conversely, *ke2* showed moderate constitutive levels that were similarly suppressed at the bag seedling stage (Figure 5G–L). These tissue-specific patterns indicate that DJ deploys robust, developmentally regulated PME machinery at the primary infection site (roots), while exhibiting differential redistribution of pectin-modifying enzymes in aerial tissues during defense activation. This deployment strategy aligns with recent findings in grapevine, where the resistant genotype ‘Souvignier Gris’ displayed significantly higher infection-induced PME activity and altered pectin methylesterification patterns compared to susceptible cultivars [37], underscoring a conserved mechanism whereby resistant genotypes actively mobilize cell wall–remodeling enzymes at the pathogen entry site to restrict fungal colonization.

Immunolocalization studies using JIM5 (Figure 6) and JIM7 (Figure 8) antibodies revealed that DJI consistently displayed significantly lower levels of both pectin epitopes in root tissues compared to controls and *ke2*I across all developmental stages. The simultaneous reduction in both JIM5 and JIM7 signals indicates active pectin depolymerization rather than simple de-esterification or epitope masking [22]. In resistant plants, this pectin degradation is not detrimental but rather serves as a controlled cell wall sacrifice to generate OGs, well-established damage-associated molecular patterns (DAMPs) that trigger immune signaling [12]. The observation that *ke2*I maintained higher levels of both pectin epitopes, despite its susceptibility, suggests that *ke2* fails to activate this DAMP-release mechanism, leaving intact pectin accessible to pathogen-derived pectinases and facilitating *Foc* 4 colonization.

### 4.2. Pectin Degradation Triggers DAMP-Mediated Immunity and PTI Amplification

The pectin degradation cascade is intimately linked to the activation of pattern-triggered immunity (PTI) [38]. Our transcriptomic data revealed massive upregulation of core PTI components in DJI, including *PTI1*, *MAPK1*, *MAPK5*, and *MAPK2* (Figure 3), indicating robust activation of the MAPK signaling cascade. Concurrently, *CDPK3* and *CML31* were strongly induced (Figure 3), connecting pectin-derived OGs to Ca^2+^-mediated defense signaling [39]. The *RBOH F*, *C*, and *C1* were also upregulated (Figure 3), driving the apoplastic ROS burst that is a hallmark of effective plant defense [40].

The contrasting regulation of *CDPK3* between cultivars is particularly informative. *CDPK3* was suppressed in *ke2* following infection but strongly induced in DJ (Figure 3). As key Ca^2+^ sensors, CDPKs directly connect cytosolic calcium signatures to the phosphorylation of downstream defense targets [41]. The specific induction of *CDPK3* in DJ, coupled with *CML31* upregulation (Figure 3), suggests that pectin-derived OGs trigger a Ca^2+^-dependent signaling hub that is defective in *ke2*. This Ca^2+^ signaling may further amplify MAPK cascades and RBOH-mediated ROS production, creating a positive feedback loop that reinforces the defense response [42].

### 4.3. Hormonal Signaling Orchestrates Systemic Defense

Phytohormones form an integrated communication network that allows plants to survey, prioritize, and launch defense operations against multiple classes of pathogens and herbivores [43]. In this regard, The PTI amplification in DJ cascades into classical hormone-mediated immunity. The bZIP transcription factor *TGA1* was specifically upregulated in DJI, but not in *ke2*I (Figure 3). *TGA1* serves as the DNA-binding partner of *NPR1*, the central regulator of SA signaling, and specifically recognizes the as-1 motif in promoters of pathogenesis-related genes such as *PR-1* [44,45]. Concordantly, a *PR1-like* gene was co-upregulated with *TGA1* in DJI (Figure 3). The coordinated TGA1–PR1 axis indicates enhanced SA-mediated immunity and systemic acquired resistance (SAR) in the resistant cultivar [46].

Beyond SA, DJI exhibited a sophisticated hormonal reprogramming signature. The JA repressor JAZ/*TIFY 10B-like* was downregulated (Figure 3), indicating derepression of JA signaling, while the ethylene response factor *ERF1B-like* was upregulated (Figure 3), reflecting ethylene–JA cross-talk essential for defense against necrotrophic pathogens [47]. The auxin-inactivating enzyme *GH3.8* was particularly upregulated (Figure 3), and auxin repressor *IAA6-like* was induced (Figure 3), indicating active suppression of auxin signaling, a hormone often exploited by pathogens to promote susceptibility [48]. This SA/JA/ET activation with auxin suppression represents the classic resistant hormonal signature.

The *MAPK5* induction in DJI (Figure 3) further supports this hormonal integration, as MAPK cascades are known to phosphorylate and stabilize WRKY and TGA transcription factors, bridging PTI to hormone-responsive gene expression [49]. Therefore, pectin breakdown in DJ sets off a hierarchical relay, progressing from OGs to Ca^2+^/MAPK/ROS signaling, subsequently activating the TGA1-SA and ERF1-JA/ET branches, and finally converging on PR gene upregulation and systemic immunity.

### 4.4. Flavonoid Biosynthesis and Secondary Defense Layer

Flavonoids serve as antimicrobial barriers, redox regulators, and immune signals that drive layered plant defense against pathogens [50]. The primary immune signaling response in DJ appears to be reinforced by a secondary metabolic defense layer. Transcriptomic analysis revealed significant upregulation of the flavonoid biosynthetic pathway in DJI (Figure 3), including key genes such as *CHI*, *CHI3*, *F3′H*, *CYP75B1*, *DFR*, and *F4S-Like*. This coordinated activation of these genes suggests enhanced production of flavonoid phytoalexins and proanthocyanidins with direct antimicrobial activity [51] against *Foc* 4. Additionally, flavonoids serve as potent antioxidants that scavenge the ROS generated during the oxidative burst, protecting plant tissues from self-inflicted oxidative damage while maintaining signaling competence [52]. The flavonoid pathway may also feed into lignin biosynthesis, contributing to cell wall reinforcement in surviving tissues after the initial pectin degradation phase [53]. The absence of comparable flavonoid pathway activation in *ke2* (Figure 3) further underscores the failure of this cultivar to mount a comprehensive defense response.

## 5. Conclusions

Our study demonstrates that *Foc* 4 resistance in banana cultivar DJ is mediated by an infection-induced, coordinated pectin degradation defense cascade that is transcriptionally activated and functionally validated in roots, suggesting preferential deployment at the primary infection site. In contrast, the susceptible mutant *ke2* lacks this precise activation, resulting in impaired defense and increased disease susceptibility. These findings highlight the central role of cell wall remodeling in banana–*Foc* 4 interactions and provide a mechanistic framework for understanding and engineering resistance to *Fusarium wilt* in banana.

## Data Availability

The original contributions presented in this study are included in the article/Supplementary Material. Further inquiries can be directed to the corresponding author(s).

## Supplementary Materials

The following supporting information can be downloaded at: https://www.mdpi.com/article/10.3390/cimb48080847/s1.
